# Triponderal mass index can be used as a potential tool to predict the risk of hyperuricemia in children and adolescents with obesity: a population-based study

**DOI:** 10.3389/fnut.2025.1544209

**Published:** 2025-07-30

**Authors:** Yang Niu, Yajie Zhang, Jinye Sheng, Yi Feng, Qingya Tang, Xiuhua Shen

**Affiliations:** ^1^Department of Clinical Nutrition, Xinhua Hospital Affiliated to Shanghai Jiao Tong University School of Medicine, Shanghai, China; ^2^Department of Clinical Nutrition, College of Health Science and Technology, Shanghai Jiao Tong University School of Medicine, Shanghai, China; ^3^Shanghai Key Laboratory of Pediatric Gastroenterology and Nutrition, Shanghai, China; ^4^Shanghai Institute for Pediatric Research, Shanghai, China

**Keywords:** hyperuricemia, triponderal mass index, waist-to-height ratio, children and adolescents, obesity

## Abstract

**Purpose:**

Hyperuricemia (HUA), a common complication in children and adolescents with obesity, has not received sufficient attention. Therefore, the purpose of this study was to compare the predictive ability of different HUA obesity indicators.

**Methods:**

The records of 349 children and adolescents with obesity aged 6–17 years (233 boys and 116 girls) who visited the Nutrition Clinic of Xinhua Hospital, Shanghai Jiao Tong University School of Medicine between January 2012 and December 2023 were included in this retrospective study. The relationship between different obesity indices and HUA was analyzed by univariate and multivariate analysis. The predictive value of triponderal mass index (TMI) and waist-to-height ratio (WHtR) for HUA was evaluated by the receiver operating characteristic (ROC) curve, and the optimal cutoff point was calculated.

**Results:**

In this study, the prevalence of HUA in the general population was 42.40% (41.20% in boys and 44.82% in girls). Multiple regression analysis revealed that after controlling for age and sex, body mass index (BMI), TMI, waist circumference (WC), hip circumference (HC), WHtR, fat mass (FM), and skeletal muscle mass (SMM) were independent risk factors for HUA (*p* < 0.05). After controlling for age and stratification by gender, BMI, WC, HC, and SMM of boys and girls with obesity were positively correlated with the risk of HUA (*p* < 0.05). However, TMI, WHtR, body fat percentage, and FM were only positively associated with the risk of HUA in obese girls (*p* < 0.05). Moreover, TMI and WHtR were 18.2 kg/m^3^ and 0.56, respectively, in the ROC curve analysis.

**Conclusion:**

The prevalence of HUA was high in children and adolescents with obesity aged 6–17 years. In addition, our results underscored that the combination of TMI and WHtR can be used as a potential early predictor of HUA risk in children and adolescents with obesity, especially in girls.

## Introduction

Hyperuricemia (HUA) is an important risk factor for diseases such as metabolic syndrome, cardiovascular disease, hypertension, type 2 diabetes mellitus, and gout (1–4). Unfortunately, HUA has become a common chronic disease among adults. However, its occurrence in children and adolescents has not received sufficient attention from clinicians and parents. It should be noted that with the increasing consumption of purine-rich meats, high fructose beverages, and the rapid rise in obesity rates, HUA has become a disease of high incidence in children and adolescents (5–8). In a study of 54,580 children and adolescents aged 3 to 19, the overall estimated prevalence of HUA was 23.3%, with a prevalence rate of 26.6% among boys, which was higher than 19.8% among girls (9). In addition, the prevalence of HUA was significantly higher in children with overweight (37.6%), obesity (50.6%), and extremely obesity (64.5%) than in normal weight children (9).

Although it has been shown that obesity increases the risk of HUA, current studies focused on which obesity indicators can effectively and rapidly predict HUA during early screening have been inconsistent. An increasing number of studies have found that traditional obesity indicators such as body mass index (BMI), waist circumference (WC), hip circumference (HC), and waist-to-height ratio (WHtR) are significantly positively correlated with HUA (10, 11). In addition, it has been shown that an increase in serum uric acid (SUA) levels leads to gradual and significant increases in BMI, WC, WHtR, percentage body fat (PBF), and muscle mass (10).

Because HUA is a common disease with obvious sex differences, we previously conducted a comparison of various obesity indicators and HUA in children and adolescents of different sex. These previous results showed that after adjusting for age, BMI, WC, HC, skeletal muscle mass(SMM), and PBF were positively associated with the risk of HUA in boys and girls with obesity (12). Although BMI, SMM, and PBF are the most influential indicators of HUA, they have shortcomings such as complex calculations and the need for specific detection equipment, which preclude them from being directly applied to early risk screening for HUA.

Recently, triponderal mass index (TMI) has attracted interest as a new tool for obesity screening in children and adolescents (13, 14). For children and adolescents aged 8–17, the TMI threshold for diagnosing overweight status is 16.0 kg/m^3^ for boys and 16.8 kg/m^3^ for girls, and the TMI threshold for diagnosing obesity status is 18.8 kg/m^3^ for boys and 19.7 kg/m^3^ for girls (13). Among children and adolescents, BMI has different diagnostic thresholds for overweight and obesity at different age groups. Its advantages include low volatility and convenient detection in different age groups. It can be seen that the advantages of TMI are not only that it changes relatively stably across different age groups, but also that it is more convenient to determine overweight or obesity. Moreover, the correlation between TMI and PBF and fat mass (FM) is much higher than BMI (13, 14).

However, currently, no studies have analyzed the relationship between TMI and HUA in children and adolescents of different sex. In the face of the increasing incidence of HUA in children and adolescents, we explored the predictive role of obesity indicators in boys and girls to provide a more concise, rapid, and reproducible method for the early detection of HUA in children and adolescents.

## Materials and methods

### Study subjects and groups

The data were collected from obese children and adolescents who visited the Nutrition Clinic of Xinhua Hospital, Shanghai Jiao Tong University School of Medicine between January 2012 and December 2023. This study was approved by the Ethics Committee of the Xinhua Hospital, School of Medicine, Shanghai Jiao Tong University (XHEC-C-2024-098-1).

The inclusion criteria for this study were (1) children and adolescents with a BMI at or above the 95th percentile (P95) for children of the same age and sex (based on WHO standards); (2) children and adolescents 6–17 years of age; (3) children and adolescents with complete data. Subjects were excluded from the study if they met one of the following criteria: (1) obesity due to endocrine or inherited metabolic diseases; (2) patients with malignant tumors and severe liver and kidney dysfunction; (3) patients taking drugs that affect uric acid levels; and (4) patients taking psychotropic drugs. The subjects were divided into an HUA group and a non-HUA group based on the SUA level of the population and stratified by male and female sex. In previous studies, HUA in children and adolescents was defined as SUA > 420 μmol/L (7 mg/dL) in boys and SUA > 360 μmol/L (6 mg/dL) in girls (9, 15).

### Anthropometric and clinical measurements

Basic participant information such as age, sex, and physical indicators were measured and recorded by a registered dietitian in the outpatient clinic. Height, WC, and HC were measured according to standard protocols. Participant body weight, PBF, FM, and SMM were measured using whole-body bioelectrical impedance analysis (InBody 720, Biospace Inc., South Korea). BMI, TMI, waist-to-hip ratio (WHR), and WHtR were calculated as follows: BMI (kg/m^2^) = (weight in kg)/(height in meters)^2^; TMI (kg/m^3^) = (weight in kg)/(height in meters)^3^; WHR = WC (cm)/HC (cm); and WHtR = WC (cm)/height (cm). Venous blood samples were obtained from fasting participants in the morning and sent to the clinical laboratory center for SUA, triglyceride (TG), and total cholesterol (TC) analysis.

### Statistical analysis

Data were analyzed using SPSS V.25.0 statistical software. Kolmogorov–Smirnov test was used to evaluate the normal distribution of parameters. Data for continuous variables are expressed as median (P25, P75), and categorical variables are expressed as frequency and percentage (%). To compare differences in various indicators between HUA and non-HUA based on sex, an independent two-tailed t test was applied for continuous data with a normal distribution, a Wilcoxon signed-rank test was used for continuous variables with a non-normal distribution, and a Chi-squared test was performed for categorical variables. Pearson correlation was used to evaluate the correlation between SUA and various factors. A multiple logistic regression model was used to evaluate the effects of different physical indicators and body composition variables on the risk of HUA. Model 1 used unadjusted regression, and Model 2 used regression adjusted for age and sex. To explore possible cutoff values for TMI and WHtR, we used receiver operating characteristic (ROC) curves to predict the occurrence of HUA. All *p*-values were calculated using a bilateral test, and the significance level of each test was set at *p* < 0.05.

## Results

A total of 349 subjects with complete data were included in the study (233 boys and 116 girls) with an average age of 10.55 years (8.69, 12.59) (Table 1). The boys were older and had higher WC, HC, WHR, WHtR, BMI, TMI, FM, SMM, and SUA than the girls (*p* < 0.05). The overall estimated prevalence of HUA was 42.4% (148/349), with 41.20% (96/233) in boys and 44.82% (52/116) in girls. WHtR values were ≥ 0.46 in the entire sample.

**Table 1:** General characteristics of study participants.

Characteristics	Total sample	Boys	Girls
*n*	349	233	116
Age, y	10.55 (8.69, 12.59)	10.97 (9.17, 12.77)	9.43 (8, 11.88)^b^
Height, cm	149.50 (138.50, 162.05)	154.40 (141.35, 165.65)	143.05 (134.17, 157.02)^b^
Weight, kg	60.50 (47.70, 79.65)	64.80 (50.40, 85.05)	52.30 (42.88, 69.55)^b^
WC, cm	89 (80.25, 98.05)	92 (84, 103.85)	84 (75.90, 90.37)^b^
HC, cm	95 (87.50, 106)	97.80 (88.95, 108)	90.50 (83, 99.75)^b^
WHR	0.93 (0.89, 0.97)	0.95 (0.91,0.98)	0.90 (0.86, 0.95)^b^
WHtR	0.59 (0.56, 0.63)	0.60 (0.57, 0.63)	0.57 (0.54, 0.60)^b^
WHtR≥0.46, *n* (%)	349 (100)	233 (100)	116 (100)
BMI, kg/㎡	27.01 (24.35, 30.22)	27.83 (25.14, 31.60)	25.32 (23.15, 28.07)^b^
TMI, kg/m^3^	18.18 (16.79, 19.41)	18.40 (16.97, 19.83)	17.70 (16.48, 18.99)^b^
PBF, %	39.25 (36.10, 42.50)	39 (35.95, 42.50)	39.78 (36.20, 43.09)
FM, kg	23.40 (18.30, 32.15)	25.30 (19.35, 33)	21.15 (15.77, 27.47)^b^
SMM, kg	19.10 (15.05, 25.65)	21.10 (15.90, 28.93)	16.24 (13.71, 21.67)^b^
SUA, umol/L	372 (311, 457)	386 (318.5, 483)	355 (307.25, 409.50)^b^
HUA, *n* (%)	148 (42.40)	96 (41.20)	52 (44.82)
TC, mmol/L	4.30 (3.86, 4.77)	4.23 (3.81, 4.70)	4.39 (3.96, 4.84)
TG, mmol/L	1.15 (0.79, 1.56)	1.18 (0.80, 1.60)	1.13 (0.76, 1.48)

WC, Waist circumference; HC, Hip circumference; WHR, Waist-to-hip ratio; WHtR, Waist-to-height ratio; BMI, Body mass index; TMI, Triponderal mass index; PBF, Percentage of body fat; FM, Fat mass; SMM, Skeletal muscle mass; SUA, Serum uric acid; HUA, Hyperuricemia; TC, Total cholesterol; TG, Triglyceride.

^b^Significance between boys and girls (*p* < 0.01).

The age, WC, HC, WHtR, BMI, TMI, FM, SMM, and TG of the HUA group were higher than those of the non-HUA group (*p* < 0.05, Table 2). In boys, age, WC, HC, BMI, TMI, FM, SMM, and TG were higher in the HUA group than in the non-HUA group (*p* < 0.05, Table 2). In girls, age, WC, HC, WHtR, BMI, TMI, PBF, FM, SMM, and TG were higher in the HUA group than in the non-HUA group (*p* < 0.05, Table 2). In the total sample, WHR was not significantly higher in the HUA group than in the non-HUA group (*p* > 0.05, Table 2).

**Table 2:** Comparison of the clinical characteristics between HUA and non-HUA groups.

Characteristics	Total sample		Boys		Girls	
	Non-HUA	HUA	Non-HUA	HUA	Non-HUA	HUA
*n*	201	148	137	96	64	52
Age, y	9.81 (8.06, 11.36)	11.94 (9.53, 14.09)^b^	10.09 (8.51, 11.45)	12.73 (10.80, 14.79)^b^	9 (7.87, 10.58)	9.73 (8.40, 12.64)^a^
Height, cm	144 (135.50, 156.35)	159.05 (145.40, 169.07)^b^	146.30 (136.90, 157.75)	166.05 (153.55, 174.15)^b^	139.50 (133.02, 148.10)	146.30 (140.95, 158.30)^b^
Weight, kg	52.90 (44.45, 66.45)	72.11 (52.12, 92.90)^b^	56.40 (46.90, 70.70)	83.20 (64.85, 102.42)^b^	48.56 (39.37, 54.49)	58.50 (50.37, 73.90)^b^
WC, cm	85.50 (77.50, 93)	94 (88, 106)^b^	88 (80, 96.75)	100.50 (91.50, 108.72)^b^	78.25 (73, 86)	88.50 (81.12, 93.37)^b^
HC, cm	99.25 (91, 108)	112.15 (101.25, 120.10)^b^	92 (86.25, 100)	105.75 (97.85, 115)^b^	85.70 (81.40, 93.87)	95.10 (89.62, 104.87)^b^
WHR	0.93 (0.89, 0.97)	0.93 (0.89, 0.97)	0.95 (0.91, 0.97)	0.94 (0.91, 0.98)	0.90 (0.86, 0.94)	0.91 (0.87, 0.96)
WHtR	0.58 (0.55, 0.62)	0.60 (0.57, 0.63)^b^	0.60 (0.56, 0.63)	0.61 (0.58, 0.64)	0.55 (0.53, 0.58)	0.58 (0.56, 0.62)^b^
WHtR≥0.46, *n* (%)	201 (100)	148 (100)	137 (100)	96 (100)	64 (100)	52 (100)
BMI, kg/㎡	25.80 (23.55, 28.86)	28.48 (26.44, 32.79)^b^	26.56 (24.14, 29.57)	30.03 (27.24, 34.36)^b^	24.58 (21.83, 26.41)	27.07 (24.11, 30.04)^b^
TMI, kg/m^3^	17.96 (16.63, 19.25)	18.41 (17.21, 19.95)^a^	18.35 (16.89, 19.46)	18.51 (17.24, 20.43)^a^	17.07 (16.19, 18.64)	18.37 (16.98, 19.07)^b^
PBF, %	39.25 (36.25, 42.10)	39.25 (35.72, 43.17)	39.30 (36.75, 42.75)	38.35 (35.42, 41.50)	38.97 (35.72, 40.85)	40.70 (37.30, 44.02)^b^
FM, kg	20.86 (17, 27.65)	27.60 (22.20, 35.97)^b^	21.90 (17.50, 28.85)	29.75 (23.32,38.37)^b^	19.05 (14.08, 22.67)	24.44 (20.19, 33.12)^b^
SMM, kg	16.80 (14.15, 22)	23.70 (16.95, 23.70)^b^	17.60 (14.95, 23)	29.30 (20.77, 36.15)^b^	15.07 (12.52, 18.36)	18.30 (14.95, 23.50)^b^
SUA, umol/L	323 (286, 356)	473.50 (430.50, 518.75)^b^	327 (284, 371)	499.50 (454, 540.25)^b^	313.50 (293, 335.75)	417 (385.25, 463.75)^b^
TC, mmol/L	4.26 (3.87, 4.67)	4.33 (3.81, 5.02)	4.23 (3.86, 4.66)	4.24 (3.69, 4.90)	4.36 (3.92, 4.77)	4.41 (4.11, 5.18)
TG, mmol/L	1.08 (0.74, 1.46)	1.31 (0.87, 1.78)^b^	1.10 (0.75, 1.51)	1.33 (0.95, 1.87)^b^	1.05 (0.73, 1.38)	1.24 (0.78, 1.76)

HUA Hyperuricemia, WC Waist circumference, HC Hip circumference, WHR Waist-to-hip ratio, WHtR Waist-to-height ratio, BMI Body mass index, TMI Triponderal mass index, PBF Percentage of body fat, FM Fat mass, SMM Skeletal muscle mass, SUA Serum uric acid, TC Total cholesterol, TG Triglyceride.

^a^Significance between HUA and Non-HUA groups in all subjects (*p* < 0.05).

^b^Significance between HUA and Non-HUA groups in all subjects (*p* < 0.01).

Correlation analysis (Table 3) showed that SUA was significantly positively correlated with age, WC, HC, WHtR, BMI, TMI, FM, SMM, and TG in the whole sample (*p* < 0.05). Further multiple regression analysis (Table 4) revealed that BMI, TMI, WC, HC, WHtR, FM, and SMM were independent risk factors for HUA after controlling for age and sex (Model 2, *p* < 0.05). In the different sex groups, after controlling for age, BMI, WC, HC, and SMM were positively correlated with the risk of HUA in boys with obesity. In addition, BMI, TMI, WC, HC, WHtR, PBF, FM, and SMM were positively associated with the risk of HUA in girls with obesity (Model 2, *p* < 0.05).

**Table 3:** Correlation analysis between clinical characteristics and SUA.

Characteristics	Total sample		Boys		Girls	
	*r*	*p*	*r*	*p*	*r*	*p*
Age	0.484	< 0.001	0.543	< 0.001	0.263	0.004
Height	0.537	< 0.001	0.579	< 0.001	0.297	0.001
Weight	0.578	< 0.001	0.604	< 0.001	0.389	< 0.001
WC	0.544	< 0.001	0.556	< 0.001	0.43	< 0.001
HC	0.534	< 0.001	0.570	< 0.001	0.362	< 0.001
WHR	0.093	0.082	0.025	0.703	0.121	0.194
WHtR	0.278	< 0.001	0.207	0.001	0.357	< 0.001
BMI	0.507	< 0.001	0.508	< 0.001	0.426	< 0.001
TMI	0.257	< 0.001	0.199	0.002	0.356	< 0.001
PBF	−0.010	0.856	−0.107	0.102	0.332	< 0.001
FM	0.488	< 0.001	0.494	< 0.001	0.402	< 0.001
SMM	0.584	< 0.001	0.614	< 0.001	0.340	< 0.001
TC	0.072	0.181	0.069	0.291	0.142	0.128
TG	0.143	0.008	0.116	0.078	0.192	0.039

SUA, Serum uric acid; WC, Waist circumference; HC, Hip circumference; WHR, Waist-to-hip ratio; WHtR, Waist-to-height ratio; BMI, Body mass index; TMI, Triponderal mass index; PBF, Percentage of body fat; FM, Fat mass; SMM, Skeletal muscle mass; TC, Total cholesterol; TG, Triglyceride.

**Table 4:** Multifactorial logistic regression analysis of the risk of HUA.

Characteristics	Model 1		Model 2	
	OR (95% CI)	p	OR (95% CI)	p
Total sample				
BMI	1.203 (1.136, 1.273)	< 0.001	1.138 (1.057, 1.225)	0.001
TMI	1.172 (1.061, 1.294)	0.002	1.130 (1.010, 1.264)	0.033
WC	1.070 (1.049, 1.091)	< 0.001	1.060 (1.030, 1.091)	< 0.001
HC	1.077 (1.055, 1.100)	< 0.001	1.062 (1.027, 1.098)	< 0.001
Each increase 0.1 in WHtR	1.189 (1.224, 2.854)	0.004	1.837 (1.128, 2.991)	0.015
PBF	0.997 (0.958, 1.038)	0.878	1.006 (0.963, 1.051)	0.784
FM	1.087 (1.059, 1.116)	< 0.001	1.049 (1.014, 1.086)	0.006
SMM	1.137 (1.099, 1.177)	< 0.001	1.175 (1.100, 1.254)	< 0.001
Boys				
BMI	1.247 (1.157, 1.344)	< 0.001	1.105 (1.010, 1.209)	0.029
TMI	1.135 (1.006, 1.281)	0.039	1.072 (0.930, 1.235)	0.338
WC	1.088 (1.060, 1.117)	< 0.001	1.038 (1.002, 1.075)	0.039
HC	1.103 (1.071, 1.135)	< 0.001	1.059 (1.017, 1.103)	0.006
Each increase 0.1 in WHtR	1.566 (0.920, 2.666)	0.099	1.147 (0.629, 2.092)	0.654
PBF	0.948 (0.902, 0.996)	0.035	0.979 (0.925, 1.037)	0.469
FM	1.110 (1.064, 1.137)	< 0.001	1.036 (0.995, 1.080)	0.089
SMM	1.176 (1.125, 1.230)	< 0.001	1.161 (1.077, 1.252)	< 0.001
Girls				
BMI	1.206 (1.082, 1.345)	0.001	1.204 (1.050, 1.380)	0.008
TMI	1.305 (1.077, 1.581)	0.007	1.264 (1.037, 1.542)	0.021
WC	1.088 (1.041, 1.138)	< 0.001	1.092 (1.034, 1.152)	0.001
HC	1.058 (1.022, 1.095)	0.001	1.071 (1.011, 1.136)	0.021
PBF	1.118 (1.032, 1.212)	0.006	1.102 (1.014, 1.197)	0.022
Each increase 0.1 in WHtR	4.141 (1.705, 10.053)	0.002	4.455 (1.732, 11.461)	0.002
FM	1.083 (1.032, 1.137)	0.001	1.088 (1.016, 1.164)	0.015
SMM	1.122 (1.041, 1.210)	0.003	1.166 (1.001, 1.359)	0.048

HUA, Hyperuricemia; BMI, Body mass index; TMI, Triponderal mass index; WC, Waist circumference; HC, Hip circumference; WHtR, Waist-to-height ratio; PBF, Percentage of body fat; FM, Fat mass; SMM, Skeletal muscle mass; OR, odds ratios.

Model 1: Crude model.

Model 2: Total sample: adjusted for age and sex; boys and girls: adjusted for age.

**Figure 1:**
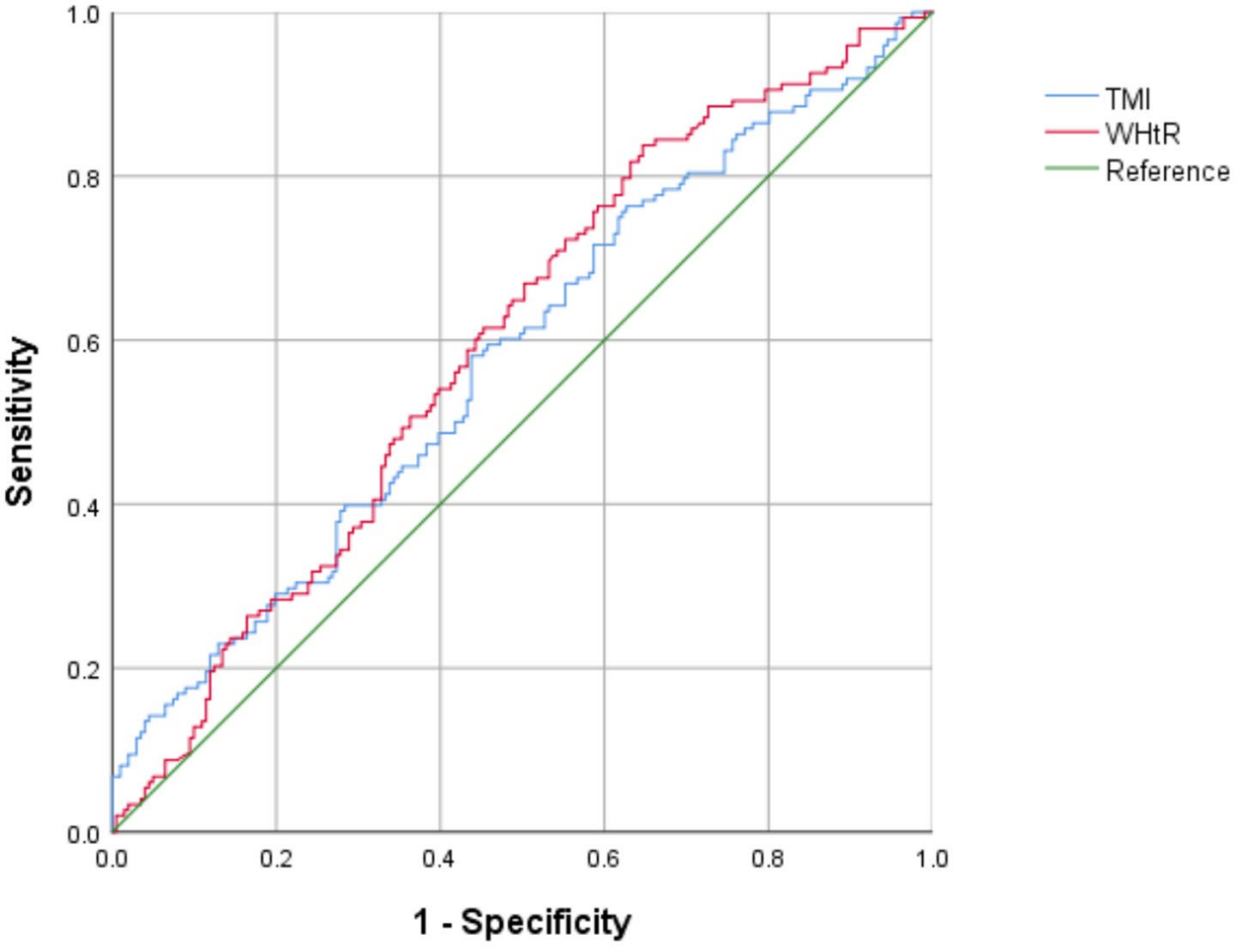
Receiver operating characteristic (ROC) curve of TMI and WHtR for identifying HUA in children and adolescents with obesity.

To further predict HUA, we performed ROC curve analysis on TMI (systemic obesity index) and WHtR (reflecting abdominal obesity) in the total sample (Figure 1). Results showed that TMI had a cut point value of 18.2 kg/m^3^ (sensitivity 58.1%; specificity 56.2%) and an area under the curve of 0.579 (95% CI: 0.518–0.639). Additionally, WHtR had a cut point value of 0.56 (sensitivity 83.8%; specificity 35.3%) and an area under the curve of 0.598 (95% CI: 0.538–0.657). Therefore, these values may be used to predict HUA (*p* < 0.05).

## Discussion

In this study, we found that the incidence of HUA in children and adolescents with obesity was as high as 42.4%. By evaluating the possibility of multiple obesity indicators as risk indicators and predictors of HUA in children and adolescents, we found that different obesity indicators had different positive effects on HUA. We also determined that TMI (18.2 kg/m^3^) and WHtR (0.56) have the potential to predict HUA.

The incidence of HUA in children and adolescents varies by country due to different diagnostic criteria, ethnicities, lifestyles, and obesity incidence. In a Brazilian study of 1,750 overweight and obese participants aged 6–17, the overall prevalence of HUA was 10.3%, and the prevalence of HUA in subjects with overweight and obesity was 51.3% (10). This is higher than the 42.4% prevalence of HUA in children and adolescents with obesity in the current study. The difference is mainly because the reference standard for HUA in the above-referenced study was lower (5.5 mg/dL) than the standards used in our study (7 mg/dL in boys and 6 mg/dL in girls) (9, 15). As the prevalence of HUA is high in children and adolescents with overweight and obesity, it is necessary to determine appropriate obesity indicators for the early prediction of HUA.

In children and adolescents, BMI and BMI-Z scores are the most commonly used indicators of obesity. In a study of 1,329 participants (16), BMI and BMI-Z scores in both boys and girls with HUA were higher than those in the non-HUA group, which is consistent with our findings. Moreover, further multiple regression analysis found that BMI and BMI-Z score were independent risk factors for HUA (16, 17). Our study further explored the influence of BMI, HC, and WC on HUA by comparing BMI with HC and WC. Results showed that the BMI odds ratio (OR) value (1.138) was higher than those of HC (1.062) and WC (1.060), and this was consistent in boys and girls.

Although BMI and BMI-Z are widely used as diagnostic indicators of obesity in children and adolescents, they cannot be quickly calculated and evaluated due to the different criteria for different ages and sex. This further limits the use of BMI and BMI-Z as routine screening indicators. Many recent studies have shown that TMI is relatively stable in different age groups, significantly positively correlated with PBF, and more accurately assesses PBF. These are advantages that BMI does not have (13, 14). Therefore, we included TMI in this study of HUA in children and adolescents. Our results suggest that TMI is an independent risk factor for HUA and that TMI OR values are higher than those of WC and HC. Because TMI is a simple and relatively stable indicator across age groups, we further used ROC curve analysis to determine the optimal TMI cutoff value based on the ability to determine the presence of HUA. Our ROC curve analysis results suggest that a TMI value of 18.2 kg/m^3^ can be used to predict the occurrence of HUA to a certain extent. In other words, when the TMI value exceeds 18.2 kg/m^3^, it should draw the attention of parents. They can consider taking the child to the hospital for a check-up and having the SUA index tested.

BMI and TMI are indicators used to assess obesity, where WC and WHtR have been proposed as simple and effective measures of central obesity (18–20). This study found that HUA was closely and positively correlated with WC and WHtR. In addition, WC was found to be an independent risk factor for HUA, which is consistent with the results of Susann et al. (21) and our previous study (12). Further ROC curve analysis showed that WHtR effectively predicted the occurrence of HUA when the cutoff value of 0.56 was reached. Regarding body composition indicators, the results showed that FM was an independent risk factor for HUA, further explaining why WHtR may play a predictive role; this was more obvious in girls. Moreover, this study found that TG was higher in the HUA group than in the non-HUA group, which may be related to higher FM in the HUA group. However, the mechanism involving the interaction between SUA metabolism, lipid metabolism, and adipogenesis remains unclear and requires further study (22).

Importantly, previous studies have shown that TMI is positively associated with an increase in WHtR in individuals aged 7–20 (23, 24). In addition, TMI has been shown to accurately identify central obesity in children and adolescents, exceeding the accuracy of BMI when WHtR is used as a central obesity reference indicator (25). Therefore, in the prediction of HUA in children and adolescents with obesity, TMI can be used as an indicator of systemic obesity for predicting the risk of HUA. This finding suggests that WHtR, as an indicator of abdominal obesity, can be combined with TMI to predict the risk of HUA.

The occurrence of HUA is not only related to obesity and FM but also to muscle mass. As muscle is a source of purines (26, 27), which plays an important role in promoting the occurrence of HUA. This explains our identification of SMM as an independent risk factor for HUA, both in the general population and in boys and girls. In addition, larger HC generally reflects higher muscle mass (28, 29), which is a potential explanation for the greater effect of HC than WC on HUA. Furthermore, our research shows that the SMM value of boys is higher than that of girls, and the OR value of SMM for boys is higher than that for girls. This result can explain to a certain extent the reason why the OR value of TMI is higher in girls than in boys.

This study has some limitations. First, this was a cross-sectional single-center study, and further multi-center prospective studies are needed to prove the relationship between different obesity indicators and HUA. In addition, indicators such as the dietary habits, physical activities, socioeconomic status and pubertal development of the participants were not included in this study. This may have caused us to overlook some HUA risk factors. Overall, obesity indicators and SUA levels can be considered a profile response to diet, lifestyle and other indicators. Third, unfortunately, blood glucose and insulin were not included in this study. In our future research, we will further conduct studies on the correlation between hyperuricemia and blood glucose and insulin. Fourth, our data were collected at a single center, which are not a representative sample of obese children and adolescents from other regions or ethnicities. In future research, we will conduct multi-center studies and further incorporate indicators such as diet, exercise, economy and genetics (30).

## Conclusion

In conclusion, the prevalence of HUA was higher in children and adolescents with obesity, but there was no difference between the sexes. In this population, BMI, TMI, WC, HC, WHtR, FM, and SMM were identified as the main risk factors for the occurrence of HUA. Furthermore, it is worth noting that considering TMI and WHtR are relatively stable and easy to calculate in all ages, they have the potential to be used as predictive indicators of HUA in children and adolescents with obesity. However, in the future, multi-center prospective studies will still be needed for further research and verification.

## Data Availability

The data that support the findings of this study are available from the corresponding author upon reasonable request. Requests to access these datasets should be directed to niuyang@xinhuamed.com.cn.
